# The role of peer communication in the socialization of adolescents' pain experiences: a qualitative investigation

**DOI:** 10.1186/1471-2431-8-2

**Published:** 2008-01-11

**Authors:** Jill E Hatchette, Patrick J McGrath, Michael Murray, G Allen Finley

**Affiliations:** 1 Interdisciplinary Research, IWK Health Centre, Halifax, Nova Scotia, B3K 6R8, Canada; 2 Research Services, IWK Health Centre, Halifax, Nova Scotia, B3K 6R8, Canada; 3 School of Psychology, Dorothy Hodgkin Building, Keele University, Keele, Staffordshire, ST5 5BG, UK; 4 Pediatric Pain Research Lab, IWK Health Centre, Halifax, Nova Scotia, B3K 6R8, Canada

## Abstract

**Background:**

Recurrent pain is a common complaint among adolescents. Children learn to resolve or cope with pain largely through family dynamics, particularly maternal influences. By adolescence, young people possess an array of pain behaviors, the culmination of multiple opportunities for modeling and reinforcement of attitudes and beliefs about pain. Adolescence is a time of increased autonomy characterized by, among other complex factors, significant increases in peer influence. Although peers are influential in health-risk behavior, little is known how peers impact adolescents' pain experience. The present study explored the role of peers in adolescents' attitudes toward pain, pain behaviors and over-the-counter analgesics.

**Methods:**

Sixty-minute focus groups were conducted with a sample 24 junior high school students from Halifax, Nova Scotia, Canada (11 male: mean age = 13.45 years, range = 12–15 years; 13 female: mean age = 13.31 years, range = 12–15 years). Participants were randomly assigned to one of five same-gender focus groups designed to explore a wide breadth and depth of information. Sessions were run until theoretical data saturation. Textual data, from transcribed audiotapes, were analyzed with the constant comparative method.

**Results:**

Peer influences were apparent in how adolescents communicate about pain and how those communications effect pain expression. Overt pain responses to injury were primarily contextual and depended on perceived threats to peer-time and pain severity. Adolescents were intolerant of peers' pain behaviors when the cause was perceived as not severe. These attitudes impacted how adolescents responded to their own pain; males were careful not to express embarrassing pain in front of peers, females felt no restrictions on pain talk or pain expression. Evidence for peer influence on attitudes toward OTC analgesics was apparent in perceptions of over-use and ease of access. Findings are discussed within the context of social information-processing and gender role expectations.

**Conclusion:**

Little research has addressed how young people experience pain within the context of the psychosocial influences that dominate during adolescence. The findings provide some insight into the role of peer influences via verbal and non-verbal communication, in adolescents' pain experience. This exploratory study is a necessary first step in understanding the socialization of adolescents' pain experiences.

## Background

Pain is a common complaint among adolescents. High prevalence of recurrent pain types such as head, stomach, ear/throat, muscle/joint/back and menstrual pain have been widely reported in the pain literature [1,2].

How adolescents learn about pain, pain behaviors and how to resolve or cope with pain is a function of many factors, most notably, the family. The intergenerational transmission of information about pain begins very early in the developing child's life from parental guidance and safety promotion to countless experienced and observed pain episodes that teach children how to respond to and cope with pain [3]. The mechanisms that account for much of how children learn about pain within the family are modeling and reinforcement. Parental modeling and reinforcement, primarily maternal, has been demonstrated within the family dynamic [4-6] and the lab setting [7,8]. With repeated incidents of pain episodes, either their own or others', children are provided with opportunities for modeling and reinforcement of attitudes and beliefs about how to experience, cope with and manage pain. A wide body of literature also supports gender differences in the socialization of pain. In an extensive critical summary of the research investigating gender variation in children's pain experiences, Unruh and Campbell reported that caregiver responses to children's pain expressions provide the children with information about social display rules and the gender appropriate responses to pain events [9]. These social display rules are maintained well beyond childhood. Research reporting significant correlations between the number of pain "models" (e.g. mother, sister, grandmother, aunt) and the frequency of pain among females suggests that social learning via observation may provide females with a supportive outlet for pain expression and numerous opportunities from which to learn socially acceptable pain responses from female peers [10].

Although the family has been well investigated in the socialization of pain, albeit still not thoroughly understood, much remains unknown about the impact peers have on the adolescents' pain experience. Adolescence is a time during which the individual experiences significant biological, cognitive, psychological and social changes that facilitate the transition from childhood to adulthood [11]. During this developmental phase, the individual begins to acquire autonomy, expand social competencies and develop identities within personal social contexts [12].

The autonomy achieved during adolescence is, in addition to other self-directed behaviors, apparent in how young people make decisions regarding their own health. By junior high school adolescents begin to take responsibility, either with or without parental assistance, for the management of their own pain [4,13-16]. Much of how adolescents experience pain can be attributed to the family, including gender differences in pain. But by adolescence, the drive toward autonomy and individuation is characterized by less time spent with parents and more time spent with peers [17].

The potentially powerful force of peers cannot be overlooked in the health-related choices made by adolescents. In a survey study, adolescents reported peers were the most influential source for health-risk behaviors [18]. Interestingly, this influence is not just manifested in the acquisition of health-risk behaviors, but in abstinence as well. Peer disapproval was associated with both marijuana and tobacco abstinence, age and peer modeling were associated with alcohol use and gender and peer disapproval were associated with sexual abstinence [18]. Clearly, adolescents' perceptions of peers' judgment can impact health behavior choices.

To date, little is known about how peers impact or develop attitudes about experience among adolescents. Guite and colleagues studied factors that influenced children's liking of a peer with pain complaints by showing 4^th ^and 5^th ^grade students vignettes of characters that had physical complaints in the presence or absence of organic disease [19]. Following the vignette, participants were required to assess the character on likeability, symptom severity and relief from responsibility (e.g. going to school). Findings indicated that children understood the difference between symptoms with and without organic disease, and perceived those with organic cause as more severe. Other findings were highly suggestive of different social role expectations for boys and girls with girls being more likely to be relieved of responsibility regardless of the presence or absence of organic cause. A negative response toward peers overtly expressing pain not considered to be severe was a clear theme among male and female adolescents in the present study.

The studies by Beal [18] and colleagues and Guite and colleagues [19] demonstrate the directional influence that social exchanges between peers can have, particularly as those exchanges relate to pain. Through social interactions, children learn adaptive social behaviors (e.g. abstinence from sexual activity, suppressing a pain response) within the context of their peer groups. The social information-processing model [20] proposes that children actively engage in a five step cognitive process when presented with social situational cues; encoding of cues, interpretation of cues, selection of desired outcome, retrieving potential responses from memory and selection of an appropriate response. This process directly impacts children's social adjustment, the extent to which children get along with peers and exhibit competent and adaptive social behaviors [21]. Within the context of the peer group, the reciprocal effects of social information-processing and the impact on social adjustment must also be considered. Throughout the course of social information-processing children can construct associations between their behavior and the outcomes of those behaviors, such as the reactions of their peers [21]. This information can then be stored in long-term memory and guide future behaviors.

Social information-processing offers a practical theoretical framework within which to understand the reciprocal nature of social interactions between peers and the subsequent behavioral responses of children. The objective of the present study was to assess adolescents' attitudes and behaviors around recurrent pain and to explore the social processes through which peers influence the development and expression of those attitudes and behaviors.

## Methods

### Design

Focus groups were chosen for this study in order to capture the breadth and depth of the adolescents' pain experiences. One of the main benefits of focus groups is that they provide insight into everyday social interaction by creating a natural environment wherein participants are influencing and being influenced by others [22,23]. Focus groups are made of individuals that are similar to each other on some dimension(s) that are of research interest. To that and, a certain amount of homogeneity is required among group members; this homogeneity can be defined either broadly or narrowly [23]. For the purposes of this particular study, it was essential to the research question that participants be young male and female adolescents that have a social structure that included full-time attendance in junior high school. Since the focus of this study was recurrent and everyday pain, there was no particular effort made to seek out participants with abnormal pain experiences. We deliberately chose two different junior high schools from neighborhoods with slightly different socio-economic statuses in order to broaden the range of opinions.

### Design Rationale

A qualitative methodology was chosen for this study for three main reasons. First, there are no known studies that specifically address the role of peers in adolescents' pain experience. For this reason, the research was exploratory in nature. Second, the present study was part of a larger study looking at the social influences of peers and family on adolescents' attitudes toward pain and pain management. Because of the paucity of data in this area, it was essential to first explore how adolescents view pain; this was best facilitated within a methodology yielding the greatest depth and breadth of information. Third, the future directions of this line of query include investigating commonalities in pain attitudes and behaviors among peers in such a way that can be generalized to a population and explained within the theoretical framework of social psychology. Since no such framework has yet to be applied to the psychosocial aspects of adolescents' pain, research questions could not be formulated *a priori *without first identifying psychosocial variables.

### Participants

Participants were 7^th^-, 8^th^- and 9^th ^grade students from a junior high school in Halifax, Nova Scotia, Canada. Approval for the study was obtained from ethics review board of Memorial University of Newfoundland, school principals and teachers. In order to obtain the fullest range of pain experiences, exclusion criteria were restricted to only the inability read or speak English and developmental disability. Three weeks before the study began, consent forms describing the study were distributed to students; parental consent and child assent were required in order to participate.

Of the 350 consent forms distributed, 36 were returned (response rate 10%). Of these, 32 adolescents agreed to participate however only 24 adolescents reported for their prearranged focus groups sessions. The final sample consisted of 11 male (mean age = 13.45 years, range = 12–15 years) and 13 female (mean age = 13.31 years, range = 12–15 years).

### Data collection

Prior to the focus groups sessions, prospective participants were randomly assigned to same-gender groups (3 male groups, 2 female groups; range of participants per group = 3–9). Those groups were then scheduled to meet at a prearranged time and location (the meeting facilities of the local mall or at their school) for the 90-minute focus groups sessions. Random assignment ensured that "cliques" would not appear in the same group and groups composed of all girls and all boys ensured the elimination of any "peacock effects" (the tendency for males to speak more frequently and authoritatively than females) and the potential for discomfort during gender-specific pain discussions. These issues were addressed at the beginning of the session and throughout. Participants were informed prior to discussions that it was essential that everyone speak and be heard and dominant talkers were managed by verbally directing attention to other group members and shy participants were drawn out with direct questions and encouragement of elaboration.

At the beginning of the focus groups session, participants were asked to fill out the Pain Incident Questionnaire, a demographic questionnaire assessing participant age, grade, gender, number of pain episodes experienced over the previous month and type, duration and intensity of pain most recently experienced. Female participants were asked additional questions regarding menstrual pain. At this point, adolescents were told that throughout the course of the upcoming discussion they should be aware that (a) there were no right or wrong answers, (b) what was discussed in the group stayed in group (with the exception of suspected self-harm), (c) any pain complaints were common and acceptable, and (d) making health decisions was a learning process at any age.

The questioning route followed explicit guidelines [22]. Conversation was elicited initially with easy questions and flowed gradually toward broad, general questions and then toward more specific questions. Focus groups were run to theoretical data saturation, the point at which no new information is forthcoming and the full range of ideas and opinions have been expressed. All sessions were audio taped and later transcribed. Participants were compensated for their time with movie video vouchers.

### Data Analysis

A trained transcriber transcribed the audiotapes of the focus groups. The data were approached with the grounded theory process outlined by [24]. Although a social influence perspective guided the theoretical framework of this study, the *a priori *consideration of themes, hypotheses and theoretical applications, was suspended until all data were analyzed. This method allowed for comprehensive consideration of all data, unconstrained by *a priori *assumptions.

The data were analyzed using a constant comparative method [25]. Transcribed audiotapes from the focus group sessions were analyzed systematically. The first step in this process was a thorough reading and re-reading, of the all of the focus group transcripts. Once the researcher was familiar with the discourse, the textual data were assessed in the form of partial and/or full sentences, as well as lengthier discourse, for similarities among participants across experiences, attitudes and opinions as they related to pain. Similar experiences, attitudes and opinions were then organized under general categories. This categorizing process was circular; themes and categories were continuously reassessed, restructured and reduced, yielding a set of textual data that best represented the most prominently emerging themes. Potential researcher bias was minimized via independent analysis of the textual data by a co-investigator. Although the study design and method of data analysis and did not lend itself to calculations of inter-rater reliability, similar conclusions were drawn regarding the themes that emerged. Differences in interpretation were typically minor and reviewed until consensus was reached.

## Results

### Prevalence of Pain

Pain types most frequently reported by adolescent males were muscle ache, joint ache and sprains. Pain types most frequently reported by adolescent females were headache/migraine, muscle ache and menstrual pain. Males reported an average of 5.18 pain episodes in the previous month. Females reported an average of 9.92 pain episodes in the previous month, almost twice as many as their male peers.

Two major thematic categories emerged from the focus group sessions: (1) peer influences on attitudes about pain and pain expression and (2) attitudes toward OTC analgesics

### Peer Influences on Attitudes About Pain and Pain Expression

#### Contextual impact

Peer influences on adolescents' attitudes toward pain, in general and in terms of their own and others' overt displays of pain behavior, were largely contextually driven.

##### Reluctance to miss activities

Adolescents reported that pain was something to be endured, particularly if there was a threat of missing extracurricular activities, e.g. "...last year, someone skated over my finger...I put gauze on it and used hockey tape to tape it up. I wanted to go back on but my coach said I couldn't...(he) wanted me to come off but I just pretended it didn't hurt. But it was hurting."

##### Fear of personal injury

This keenness to participate in valued social activities was balanced by a personal assessment of the seriousness of the pain. In situations where pain was perceived as extreme or unfamiliar, adolescents recognized the ramifications of further damage, e.g. "I know if I play (with a sprained finger), I'll hurt myself or whatever or make it worse."

#### Empathy and tolerance

Adolescents tended to legitimize their peers' pain expression in terms of whether they perceived that pain to be real or not. Clear distinctions were made between pain that was real and pain that was likely minor but exaggerated for the sake of attention-getting. Overall, adolescents expressed negative attitudes toward peers who complained about pain, e.g. "Well the real pain is pain that everyone would feel. But 'wussy' pain is something that one person would whine about and the other person would just sort of live with it."

#### Pain discussion among peers

Attitudes about pain were also apparent in how adolescents spoke to each other about pain experiences. For females, pain was just another topic of conversation – little attention was paid to censoring ones' experiences or limiting discussions within gender, e.g. Moderator: "But you guys don't mind talking to each other about it (pain)?" Female Adolescent: "No" Moderator: "What about talking to boys about it?" Female adolescent: "No, we talk about it all the time...pretty much tell anybody, like we don't really care. We're not like – 'we can't tell the boys' – because they understand"

For male adolescents, pain talk was largely within gender and the context of pain experiences largely influenced the extent of discussion. Although boys talked about pain with other boys, they were careful that they were not perceived as being soft, e.g., "It depends...like if my friends are snowboarding and you go up to do a trick and you fall? I just get back up and make it look like it didn't really hurt. But with basketball, if I got hit in the head with a ball, and it actually hurt, I'd have to go off. I wouldn't be like 'Oh, it doesn't hurt"'

### Attitudes toward OTC analgesics

Although adolescents reported using OTC analgesics for the treatment of pain, attitudes toward OTC analgesics varied from negative to positive.

#### Negative

Adolescents' concerns about OTC analgesics generally centred on their chemical composition, ill effects and dependence, e.g. "...people are still working to find out stuff about them and see if they're bad for you or good for you. Most of the time, they're not good at all." and "Some people take too much medication. Some of my friends have (ibuprofen) and they just take it for everything. They don't wait and see if their headache goes away or wait and see if they're just over-reacting or something. They just automatically take it."

#### Positive-cautious

Although some adolescents expressed concern about OTC analgesics, others recognized the necessity of using medications for pain management, e.g. "I'd rather have no pain for a little bit, than have to NOT take pain killers and have pain all the time."

#### Positive

Other adolescents expressed positive attitudes toward OTC analgesic use and indicated that having medications on their person was standard practice, e.g. "...I take a bottle (of ibuprofen from home) and keep it in my bag." and "Everyone has it if you need it."

## Discussion

### Adolescent Pain Types

The pain types reported by adolescents during focus group discussions were consistent with those reported in the adolescent pain literature [1,2]. Gender differences were apparent in reported pain types as well as reported pain frequency with female adolescents reporting more varied pain types and more frequent occurrences of pain than male adolescents. Gender differences in reported headache and migraine may be attributable to the rise and increased fluctuation of hormone levels in young girls during puberty [26]. In addition to biological differences, differences in social display rules may account for gender differences in reported pain types. Where young girls are socialized to express pain more openly than young boys, the female adolescent in the present study may have felt more at ease reporting all pain types experienced [27].

### Attitudes About Pain and Pain Expression: The Influence of Peers

How adolescents coped with pain was often contingent on peer-related events and the potential for pain to interfere with participation in those events. The adolescents participating in this study had a basic understanding of the physiology of pain and understood the necessity of responding to and resolving pain. However, practical responses to the possibility of further injury were conflicted with the desire to continue participating in extracurricular activities with peer. Given the importance of peers during this developmental phase, it is not surprising that minor pain complaints would be concealed in order to secure time that had been arranged to spend with peers [17]. These responses can be understood within the context of the social information-processing model. Based on participants' responses, it was apparent that cognitive processes were engaged yielding a behavioral response; situational cues were encoded (e.g. an injury is apparent and peer time is being threatened), interpreted (e.g. there is a chance of being removed from the game), a goal set (e.g. to not be removed from the game), potential responses assessed (e.g. hide pain and stay in game or admit pain and leave game), choose most positively evaluated response (e.g. stay in game since injury is not severe or leave game since injury is severe).

Perhaps the most interesting peer influence to emerge in the focus groups discussion was the impact that *peers*' pain expression and behaviors had on adolescents. Adolescents possessed particularly negative attitudes about pain in the context of peer pain expressions. In particular, adolescents were intolerant of peers' overt expression of pain that was not perceived to be severe. Similar findings have been reported among school-aged children [19]. Lack of empathy for peers expressing minor pain may have an impact on how adolescents talk to their peers about their own pain experiences. This finding underscores the importance of reciprocity in social interactions between peers. The feedback that adolescents' get from their immediate peer group can have a huge impact on how future behavioral responses are chosen. The way in which a peer responds to a child's behavior (e.g. either negatively or empathetically to pain behaviors) may affect self-perceptions, which in turn may impact social adjustment [21].

Gender differences were apparent in how adolescents communicated about pain. Where females were likely to talk about their pain "all the time", males were more likely to use qualifiers; in particular, adolescent males tended to minimize pain that was the result of their own mishap, particularly when this pain occurred in the presence of their peers. For the adolescents in the present study, females felt more freedom to discuss all types of pain since most of the time there was an organic cause. However, males were more likely to distinguish between pain types regardless of organic cause, only discussing "real" pain (that with a legitimate social cause, as opposed to a legitimate organic cause) and remaining quiet about pain resulting from mishap or clumsiness.

The reluctance to speak about pain that is perceived to be less than legitimate (i.e. embarrassing) may be best understood by gender role expectations and social display rules. For example, social display rules for anger, sadness and pain expression may depend largely on who is present [27]. Children are more likely to moderate emotional responses in front of their peers than in front of their parents. This is particularly true for boys who may not feel the same freedom and support that girls do to express pain. Males may be cautious about expressing pain behaviors among their peers so that they do not appear to be weak. Conversely, females are socialized to be supportive about pain, express pain behaviors and openly discuss pain [10,27].

### Attitudes About Pain Management

Most of the adolescents participating in the present study had, at some time, self-medicated for recurrent pain. Consistent with the existing pain literature, females were more likely to self-medicate for pain, most of which was headache and menstrual pain [7,14,15]. Adolescents expressed a wide range of attitudes toward OTC analgesics. Although negative attitudes were expressed, they did not appear to be based on personal experience or scientific evidence. Nor did negative attitudes seem to affect the pharmacological pain management strategies chosen by adolescents. Negative attitudes may be due, in part, to the heavy exposure adolescents experience about the dangers of illicit drugs [28]. Negative attitudes about illicit drugs may possibly transfer to negative attitudes about all medications.

Where peer influences in OTC analgesic use were considered, there were clear gender differences in medication access and availability. Most notably, females were more likely to carry OTC analgesics in their backpacks and share them with their friends because "we get menstrual pain." The "norm" for girls to carry OTC analgesics to school may be related to analgesic use persisting beyond the normal duration of discomfort [29] and the generalization of menstrual pain complaints to other types of pain [13]. However, school policy may likely be influential in adolescent girls' choice to carry OTC medications to school; in both of the schools participating, teachers or administrators could not provide children with OTC analgesics. Although peer influence in OTC analgesic use was apparent for female adolescents in the present study, there was no clear indication that peers were influential in pain management choices in general. The primary social influence in adolescents' pain management choices appears to be parental, specifically maternal, and has been described elsewhere [4].

## Conclusion

The current study provides a descriptive picture of the social influences in adolescents' recurrent pain experience. To date, no known research has addressed the rich social context within which adolescents experience recurrent pain. Although peer influences in the acquisition of risky health behaviors have been well documented little research has been conducted on peer influences in adolescents' attitudes toward recurrent pain, pain expression and pain management. The data from the current study indicates that peers are essential facilitators in the development of attitudes about pain and in particular, pain expression.

## Study Limitations

Low response rates and a subsequently small sample was the greatest limitation of the current study and warrant further consideration. Qualitative methodologies necessarily require participants to commit significant time and energy to participate in focus groups sessions. Adolescents' time, in particular, may be difficult to secure once school has been concluded for the day. For example, all of the twelve participants who failed to report to scheduled focus group sessions had been seen by classmates leaving the school with friends. Another issue that should be considered is how traditional methods of recruiting adolescents (e.g. from schools) for research studies are ineffective. For example, consent forms distributed at schools may never be shown to parents, school announcements and reminders may not be heard, free pizza and a $10 movie voucher may not be incentive enough to participate in a focus group. The poor response rate in the present study merits reconsidering the ways in which we reach and engage this particular age group.

It is important to note however, that the purpose of a qualitative study is to describe the social reality of a particular group at a particular time – generalization or "transferability" is not the ultimate goal. Nevertheless, the final sample was clearly a disproportionate representation of individuals for whom the subject matter was of particular interest, who possessed very salient attitudes about pain and pain management or who simply were more organized than those who did not participate. These characteristics are not likely representative of the general population making the generalization of conclusions difficult to make beyond the study sample that was employed.

## Future Directions

Given the evidence for the salience of peers and social context on the adolescents' pain experience, future directions in this area include more in depth investigations of the decision making processes that impact adolescents' pain behaviors via social information-processing. In addition, the qualitative data obtained from the present study can be used to inform experimental designs investigating the impact of peer influence on adolescents' pain behavior and the influence of peers on pain attitudes and pain expression among adolescents with chronic pain conditions. The clinical implications for the further development of this research include a better understanding of the social factors that impact how adolescents interpret and express pain within the context of peer relations and using that knowledge to provide better supportive resources for adolescents with chronic pain. For example, the development and of web-based intervention as a vehicle for support among adolescents with chronic pain conditions could have the potential to provide peers with a safe environment to share pain experiences, facilitate adaptive coping strategies and potentially moderate disability.

## Competing interests

The author(s) declare that they have no competing interests.

## Authors' contributions

JH was responsible for conception and study design, ethics preparations, participant recruitment, focus group moderation, data analysis and manuscript preparation.

PM was principal investigator of the Canadian Institutes of Health Research (CIHR) grant which funded this study, and also responsible for critical appraisal of study design, ethics preparation and manuscript preparation.

MM was co-principal investigator of the CIHR grant which funded this study and also responsible for critical appraisal of the research design and data analysis as well as manuscript preparation.

GAF contributed to intellectual content and critical appraisal of research design, ethics preparation and manuscript preparation.

All authors have read and approve the final version of this manuscript.

## Pre-publication history

The pre-publication history for this paper can be accessed here:


